# Flavonoid Fraction of Orange and Bergamot Juices Protect Human Lung Epithelial Cells from Hydrogen Peroxide-Induced Oxidative Stress

**DOI:** 10.1155/2015/957031

**Published:** 2015-06-21

**Authors:** Nadia Ferlazzo, Giuseppa Visalli, Antonella Smeriglio, Santa Cirmi, Giovanni Enrico Lombardo, Pietro Campiglia, Angela Di Pietro, Michele Navarra

**Affiliations:** ^1^Department of Drug Sciences and Products for Health, University of Messina, Viale Annunziata, 98168 Messina, Italy; ^2^Department of Biomedical Sciences and Morphological and Functional Images, University of Messina, Via Consolare Valeria, 98100 Messina, Italy; ^3^Department of Pharmaceutical and Biomedical Sciences, University of Salerno, Via Ponte don Melillo, Fisciano, 84084 Salerno, Italy

## Abstract

It has been reported that oxidant/antioxidant imbalance triggers cell damage that in turn causes a number of lung diseases. Flavonoids are known for their health benefits, and *Citrus* fruits juices are one of the main food sources of these secondary plant metabolites. The present study was designed to evaluate the effect of the flavonoid fraction of bergamot and orange juices, on H_2_O_2_-induced oxidative stress in human lung epithelial A549 cells. First we tested the antioxidant properties of both extracts in cell-free experimental models and then we assayed their capability to prevent the cytotoxic effects induced by H_2_O_2_. Our results demonstrated that both *Citrus* juice extracts reduce the generation of reactive oxygen species and membrane lipid peroxidation, improve mitochondrial functionality, and prevent DNA-oxidative damage in A549 cells incubated with H_2_O_2_. Our data indicate that the mix of flavonoids present in both bergamot and orange juices may be of use in preventing oxidative cell injury and pave the way for further research into a novel healthy approach to avoid lung disorders.

## 1. Introduction

The functional properties of the respiratory apparatus make it particularly susceptible to oxidative stress, coming mainly from inhaled prooxidant compounds present in airborne pollutants, working environments, and cigarette smoke [1]. These pollutants trigger the overproduction of free radicals, such as reactive oxygen species (ROS). This leads to oxidative damage often linked to the etiopathogenesis of several chronic lung disorders, including chronic obstructive pulmonary disease (COPD), asthma, and adult respiratory distress syndrome (ARDS) [2]. Chronic respiratory diseases have a widespread and rapidly growing public health impact (affecting hundreds of millions of people worldwide) and consequently have a high economic impact and social cost [3]. The complex interactions between environmental and genetic factors occurring in lung diseases cause the activation of proteolytic enzymes, lipoxygenase and cyclooxygenase which in turn produce large quantities of ROS leading to pulmonary inflammation that is responsible for the progressive (and only partially reversible) restriction of lung airflow [4, 5].

In recent years there has been an extraordinary increase in the number of studies on various phytochemicals due to their antioxidant properties that enable them to counteract ROS overproduction. Among these, flavonoids, a family of polyphenols found especially in fruits, vegetables, red wine, and tea, have been extensively studied [6, 7]. Flavonoids are plant secondary metabolites commonly found in the fruits and vegetables regularly consumed by humans. Their antioxidant activity ameliorates many inflammatory diseases and has linked to the maintenance of good health [8, 9]. Several mechanisms are involved in the beneficial effects exerted by flavonoids, including the free radicals scavenging [10], the transition metal ions chelation [11], the enhancement of glutathione content, and modulation of defense genes expression via the Nrf2/ARE pathway [10, 12–14].* Citrus* fruits and their juices are the main food sources of flavonoids and have been extensively studied as regards their anticancer, cardiovascular, and anti-inflammatory activity [15].

The health properties of* Citrus sinensis* (orange) have long been studied [16], while scientific interest in* Citrus bergamia* (bergamot) derivatives has gained ground only in recent years [17, 18]. We have recently reported the anticancer properties of bergamot juice (BJ) in different* in vitro *[19] and* in vivo* models [20] and proposed the flavonoid fraction of BJ (BJe) to be responsible for this action [21]. We have also shown that low concentrations of BJe reduce the LPS-induced inflammatory response in THP-1 monocytes through SIRT1-mediated NF-*κ*B inhibition [22] and exert an anti-inflammatory effect* in vivo *[23].

The drugs currently available have proved poorly effective in treating or preventing airway diseases. Therefore, novel preventive and therapeutic approaches that target primarily the causative mechanisms of these diseases are needed. On the basis that oxidative stress plays a key role in the pathogenesis and progression of lung disorders, the use of antioxidants should be given a higher profile. In this regard, several studies have shown a beneficial links between fruit and vegetables or natural antioxidant intake and lung diseases [24, 25]. Indirect epidemiological evidence reports a positive correlation with lung function and a negative one between apple intake and asthma prevalence and incidence suggesting that flavonoids might protect against COPD [26, 27]. These findings are consistent with other research reporting that fruit consumption is negatively correlated with incidence of chronic nonspecific lung disease, prevalence of COPD symptoms, and asthma, thus improving lung function [28, 29]. Although the antiallergic and anti-inflammatory properties of flavonoids might explain their beneficial effects observed in asthma [27, 30], their real clinical effectiveness has yet to be established.

The present study was designed to evaluate the effect of the flavonoid fraction of both bergamot and orange juices against oxidative stress in human lung epithelial A549 cells.

## 2. Material and Methods

### 2.1. Biochemicals and Reagents

All chemicals and reagents were obtained from Sigma-Aldrich (Milan, Italy) unless otherwise specified.

### 2.2. Orange and Bergamot Juice Extracts

The flavonoid fraction of both orange and bergamot juices (FFOJ and FFBJ, resp.) were provided by the company “Agrumaria Corleone” (Palermo, Italy). The fruits of* Citrus bergamia* Risso & Poiteau (bergamot) came from crops located in the province of Reggio Calabria (Italy), while those of* Citrus sinensis* var. Tarocco (orange) were from groves situated in Eastern Sicily (Italy). The extracts were centrifuged, transformed into a powder by spray drying, and then stored at −20°C. Finally, they were defrosted, diluted in culture medium, pH adjusted to 7.4, and filtered immediately prior to use.

### 2.3. Chemical Characterization of the Juices Extracts

Qualitative and quantitative composition of FFBJ and FFOJ were determined using previously described protocol [21, 22]. Briefly, both FFBJ and FFOJ were solubilized in methanol to a concentration of 1 mg/mL^−1^, ultrasonicated and filtered through a 0.2 *μ*m nylon membrane (Millipore, Milan, Italy), and then injected into a UHPLC coupled online to an LCMS-IT-TOF mass spectrometer (Shimadzu, Kyoto, Japan). Identification of flavonoids was carried out on the basis of diode array spectra, MS molecular ions, and MS/MS fragmentation patterns. Data obtained were compared with those available in scientific literature. Molecular formulae were calculated by the Formula Predictor software (Shimadzu).

### 2.4. Evaluation of Antioxidant Capacity

#### 2.4.1. Folin-Ciocalteu Method

The total phenolic contents of FFBJ and FFOJ were determined by means of the Folin-Ciocalteu-assay, following Tomaino et al. [31]. Briefly, 50 *μ*L of methanol/water solutions of different sample concentrations was added to 450 *μ*L of deionized water, 500 *μ*L of Folin-Ciocalteu reagent, and 500 *μ*L of 10% sodium carbonate solution and incubated in the dark at room temperature for 1 h, vortexing every 10 min. Absorbance was recorded at 786 nm (Prixma UV-Vis Spectrophotometers) against a blank containing 50 *μ*L of the same solvent used to dissolve the extracts. Total phenol content is expressed in mg of gallic acid equivalents (GAE/g of dried extract).

#### 2.4.2. Quenching of the Stable 2,2-Diphenylpicrylhydrazyl (DPPH) Radical

The DPPH assay was used to evaluate the radical scavenging activity of FFBJ and FFOJ. Following the procedure devised by Tomaino et al. [31], different concentrations (ranging from 0.1 to 1 mg/mL) of methanol/water solution of each extract or vehicle alone (37.5 *μ*L) were added to 1.5 mL of DPPH methanolic solution (25 mg/L). Absorbance was measured at 517 nm 30 min after starting the reaction. Free radical scavenging capacity of juice extracts is expressed in mg of Trolox equivalents (TE/g of dried extract).

#### 2.4.3. Oxygen Radical Absorbance Capacity (ORAC) Assay

Antioxidant activity of FFBJ and FFOJ against 2,2′-azobis(2-amidinopropane) dihydrochloride (AAPH) peroxyl radicals was chemically examined using the ORAC method described by Dávalos et al. [32] with some modifications. Briefly, several concentrations of FFBJ or FFOJ (20 *μ*L) in 75 mM phosphate buffer solution (pH 7.4) were mixed with 120 *μ*L of 417 nM fluorescein solution and incubated at 37°C for 15 min to which 60 *μ*L of AAPH (40 mM) was then added. Fluorescence was recorded spectrofluorometrically every 30 sec for 90 min (*λ*_ex_ 485; *λ*_em_ 520; FLUOstar Omega, BMG Labtech), and the decrease in fluorescence was monitored. A blank, using phosphate buffer instead of sample, and calibration solutions of Trolox (10–100 *μ*M) were also included in each assay. The ORAC value was calculated using the area under the fluorescence decay curves and is expressed in *μ*moles of TE/g of dried extract.

#### 2.4.4. Reducing Power

The reducing power of FFBJ and FFOJ was determined following the method described by Martorana et al. [33]. In brief, 0.2 mL of several concentrations of extracts was mixed with 0.5 mL of 0.2 M sodium phosphate buffer (pH 6.6) and 0.5 mL of 1% K_3_Fe(CN)_6_ and then incubated in a water bath at 50°C for 20 min. Subsequently, 0.5 mL of 10% TCA was added to the mixture which was centrifuged at 8300 ×g for 10 min. The supernatant (0.5 mL) was then mixed with 0.5 mL of distilled water and 0.1 mL of 0.1% ferric chloride solution and absorbance measured at 700 nm. Increased absorbance of the reaction mixture indicated increased reducing power. Ascorbic acid was used as a reference. Phosphate buffer was used as blank solution. Reducing power is expressed in mg of ascorbic acid equivalent (AAE)/g of dried extract.

### 2.5. Cells Culture Conditions and Treatment

The biological experiments were performed using a basal epithelial cell line A549 derived from human lung carcinoma (ATCC, Rockville, MD, USA). Cells were grown in 6-well plates (approximately 3 × 10^5^ cells/well) and cultured in RPMI medium with 2 mM L-glutamine (Gibco Invitrogen, Milan, Italy), 10% (v/v) foetal bovine serum (FBS), 100 IU mL^−1^ penicillin, and 100 g mL^−1^ streptomycin at 37°C in a humidified 5% CO_2_ atmosphere. When 80–90% confluence was reached, monolayers were used for experiments by adding FFBJ and FFOJ to obtain a final concentration of 25 and 50 *μ*g mL^−1^ in cell medium with 2% FBS. After 18 h, the medium was removed, and cells were washed and exposed to 200 *μ*M H_2_O_2_ in phosphate buffer saline (PBS) solution (pH 7.4) containing 10 mM D-glucose for a further 2 h.

For each set of experiments, a negative control (untreated cultures) and a stressor control (H_2_O_2_ alone) were prepared by replacing the extracts with PBS.

### 2.6. Cytofluorimetric Analyses

Fluorescence-activated cell sorting (FACS) techniques were employed to determine the following parameters: intracellular ROS, lipid hydroperoxides, 8-oxo-7,8-dihydro-2′-deoxyguanosine (8-oxo-dG), transmembrane mitochondrial potential (Δ*ψ*_m_), and cell viability. After each experiment, the cells were harvested, centrifuged at 1000 ×g for 5 min, and washed and suspended in PBS. Aliquots of cell suspensions (~2 × 10^5^ cells mL^−1^) were used for each probe as described below.

The lipophilic membrane-permeable 2-7-dichlorofluorescein diacetate (DCF-DA, 1 *μ*M) was used as probe to evaluate intracellular ROS. The molecule, which undergoes deacetylation by intracellular esterases, is rapidly oxidized in its highly fluorescent derivative 2′-7′-dichlorofluorescein (DCF) in the presence of ROS. The loaded cell suspensions were incubated at 37°C for 30 min and the fluorescent signals were collected in the FL-1 channel (530 ± 20 nm) [34].

Lipid hydroperoxides were detected using the diphenyl-1-pyrenylphosphine probe (DPPP; Invitrogen Molecular Probe, Milan, Italy) as previously reported [34]. The probe reacts stoichiometrically with lipid hydroperoxides in cell membranes to yield a fluorescent phosphine oxide (DPPP=O) and the corresponding hydroxide. DPPP was added to cell suspensions to obtain a final concentration of 150 *μ*M and incubated at 37°C for 3 h. Fluorescent phosphine oxide signals were then collected in the FL-1 channel.

The fluorescent probe rhodamine 123 (R123; Invitrogen Molecular Probes) was used to assess the Δ*ψ*_m_ due to its ability to cross the mitochondrial membrane and accumulate in the matrix of functional mitochondria. The fluorochrome R123 (10 *μ*M) was added to the cell suspensions and incubated at 37°C for 10 min. Signals were collected in the fluorescence channel above 600 nm (FL-2 channel) [35].

Levels of 8-oxo-dG were measured using the FITC-labelled avidin probe to assess oxidative DNA damage [21]. This technique is based on the high affinity of avidin to 8-hydroxyguanine (8-OH-Gua) due to the remarkable structural similarities between the hydroxylated form (8-OH-Gua) and biotin. The probe binds with high specificity to 8-oxo-dG and does not require sample pretreatment for DNA isolation and hydrolysis, thus reducing artifactual oxidation [36]. In detail, cells permeabilised with methanol (15 min at −20°C) were incubated with avidin-FITC conjugate (0.2 *μ*M) at 37°C for 1 h, and then the fluorescence was collected in the FL-1 channel. The emission values detected in untreated cells correspond to 4.5 ± 1.78-oxo-dG/10^7^ dG, which is the background level of oxidised base in A549 [37].

Cell viability was evaluated by adding propidium iodide (3 *μ*g mL^−1^) to cell suspensions at 4°C for 3 min. Dead cells, stained with the DNA intercalating probe, were counted by measuring emission signals in the FL-3 channel.

The data collected from each probe were used to draw the respective curves by calculating the average of cell percentages for each emission value. In FACS analyses, the weighted average of emission values* per* 100 cells was calculated and is expressed in arbitrary fluorescence units (AFU). The values obtained were used to calculate the percentage changes (%Δ) compared to the respective control.

### 2.7. Comet Assay

Cells treated as reported above were assayed for DNA integrity by the alkaline version of the comet assay following Picerno et al. [38]. Tests were performed in duplicate on about 2 × 10^4^ cells* per* spot, and electrophoresis was carried out at 300 mA and 25 V (0.86 V cm^−1^) for 30 min. The slides, stained with ethidium bromide (20 *μ*g mL^−1^), were analysed within 24 h at 400x magnification under a DMIRB fluorescence microscope (Leica Microsystem Heidelberg GmbH, Mannheim, Germany), equipped with a digital camera (Canon, Milan, Italy).

One hundred randomly selected nuclei were acquired for each coded spot and underwent automated image analysis CASP. The following parameters were considered: tail length (TL), percentage of DNA in the tail (TDNA%), and tail moment (TM).

### 2.8. ROS and Δ*ψ*_m_ Determinations by Confocal Microscopy Observations

A549 cells were grown on cell chamber slides and treated as described above. The two probes DCF-DA and R123 were used separately to load cells. Treated and untreated cells were observed using TCS-SP2 confocal laser scanning microscopy (CLSM) equipped with an Ar/Kr laser (Leica Microsystems, Germany).

### 2.9. Statistical Analysis

All data are presented as mean ± SEM based on at least three independent experiments. Significance was set at *P* < 0.05. Comparisons and correlations were calculated using one-way analysis of variance (ANOVA) and Pearson's correlation coefficient, respectively.

## 3. Results

### 3.1. Chemical Composition of FFBJ and FFOJ

**Figure 1:**
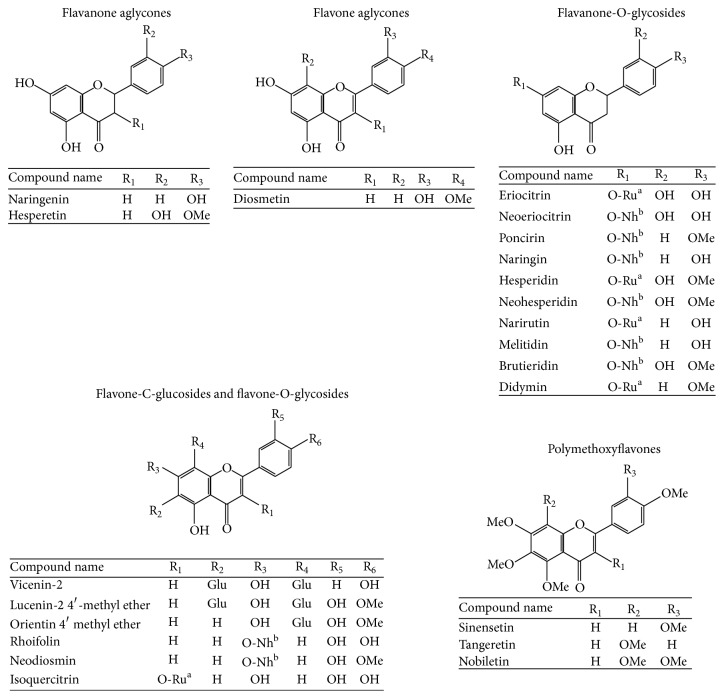
Chemical structures of flavonoids found in FFBJ and FFOJ. ^a^Rutinose (Ru). ^b^Neohesperidose (Nh). Methyl group (Me). Glucoside (Glu).

The chemical composition of the FFBJ and FFOJ extracts used in this study are shown in Table 1 and expressed in mg/g of dried extract.  Figure 1 shows the chemical structures of the flavonoids detected. As expected, neohesperidin, naringin, hesperetin, and melitidin were the main compounds present in FFBJ, although neoeriocitrin and naringenin were also present in quite large quantities. In FFOJ the highest amounts of flavonoids present were hesperidin, narirutin, and vicenin-2. Although both extracts belong to the* Citrus* family, FFBJ and FFOJ differed regarding their abundance in flavonoids and the relative percentages (Table 1). For instance, some compounds present in FFBJ were not present or undetectable in FFOJ, while hesperidin and narirutin were abundant in FFOJ and scarce in FFBJ. A slight difference in chemical composition may reflect significant differences in both physicochemical properties and biological activities.

### 3.2. Antioxidant Capacity in Cell-Free Models

The antioxidant and radical scavenging properties of FFBJ and FFOJ were determined using a range of tests that highlighted significant differences between the two* Citrus* juice extracts. As shown in Table 2, total phenolic compounds content, measured by the Folin-Ciocalteu method, was higher in FFBJ than in FFOJ (*P* < 0.05). This was directly correlated to the overall reducing capacity of a sample measured by the Reducing Power test. The latter assay confirmed a significant difference between FFBJ and FFOJ (*P* < 0.001). Moreover, antioxidant capacity, evaluated by ORAC assay, confirmed the significant differences between the two extracts (*P* < 0.01), while no statistically significant differences were detected by the DPPH^∙^ test.

### 3.3. Biological Assessment

**Figure 2:**
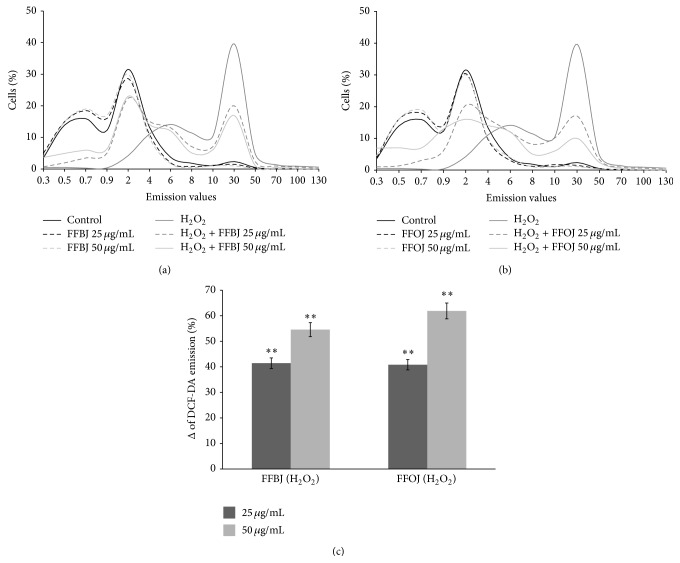
Cytofluorimetric evaluation of intracellular ROS. A549 cells treated for 18 h with FFBJ or FFOJ were oxidatively stressed by H_2_O_2_ 200 *μ*M for additional 2 h. Results from FFBJ (a) or FFOJ (b) treatments. The curves shifted rightward to higher emission values indicate the increase of ROS production. Data from (a) and (b) are expressed as percentage of reduction (%Δ) of DCF-DA emission values in FFBJ or FFOJ-pretreated cells and then exposed to H_2_O_2 _compared to H_2_O_2_-stressed cells (c). The experiments were repeated at least three times. ^*∗∗*^ *P* < 0.01* versus* H_2_O_2_-treated cells.

**Figure 3:**
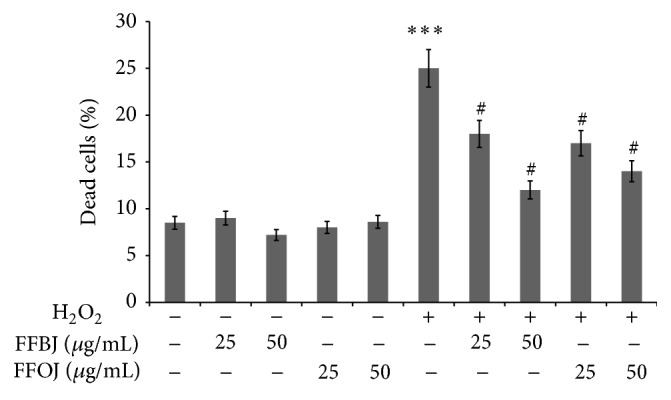
Effect of FFBJ or FFOJ on cell death induced by H_2_O_2_. The cells were treated for 18 h with FFBJ or FFOJ followed by incubation with 200 *μ*M H_2_O_2_ for additional 2 h. PI-positive cells were determined by flow cytometry collecting the emission signal in the FL-3 channel. Data represent means ± SEM of three separate experiments. ^*∗∗∗*^ *P* < 0.001* versus* control cells and ^#^ *P* < 0.05* versus* H_2_O_2_-treated cells.

To study the potential protective effects of both FFBJ and FFOJ in different compartments of oxidatively injured A549 lung epithelial cells, we first measured the intracellular content of ROS and checked cell viability in H_2_O_2_-stressed or unstressed cells, which had either been pretreated or not with* Citrus* extracts. As expected, DCF emission values in cells treated with FFBJ and FFOJ for 18 h did not differ significantly from the background values recorded in untreated cells (Figure 2). Similarly, no differences between FFBJ- or FFOJ-treated and untreated cells were recorded in PI emission values (Figure 3). These results indicate that the extracts at both 25 and 50 *μ*g/mL concentrations did not trigger ROS generation or affect cell viability (always >90%). Instead, DCF emission values in H_2_O_2_-stressed cells were up to 6.7-fold higher than those detected in untreated cultures, suggesting that there had been an increase in ROS generation (Figure 2). Consequently, H_2_O_2_ caused 25% cell death, as detected by FACS analysis (Figure 3). Interestingly, as shown in Figures 2(a) and 2(b), the presence of* Citrus* extracts reduced H_2_O_2_-induced oxidative stress, thus preventing the increase in ROS. Indeed, the fluorescence emission curve for untreated culture shows a consistent peak (proportional to the number of cells with low emission values) on the left of the graph. In contrast, in A549 cells incubated with 200 *μ*M H_2_O_2_ for 2 h a larger number of cells with high emission values were detected (the gray peak on the right), indicating an increased ROS production.

The curves obtained from the cells pretreated with* Citrus* extracts and then incubated with H_2_O_2_ show a smaller number of cells with high emission values.  Figure 2(c) summarizes data from the cytofluorimetric analyses shown in Figures 2(a) and 2(b), showing the percentage (%Δ) of ROS reduction in A549 cells pretreated with FFBJ or FFOJ and then incubated with H_2_O_2_ in comparison to the cells exposed to H_2_O_2_ alone. The histograms illustrate that both extracts significantly reduced ROS production caused by H_2_O_2_ in A549 cells (*P* < 0.01).

**Figure 4:**
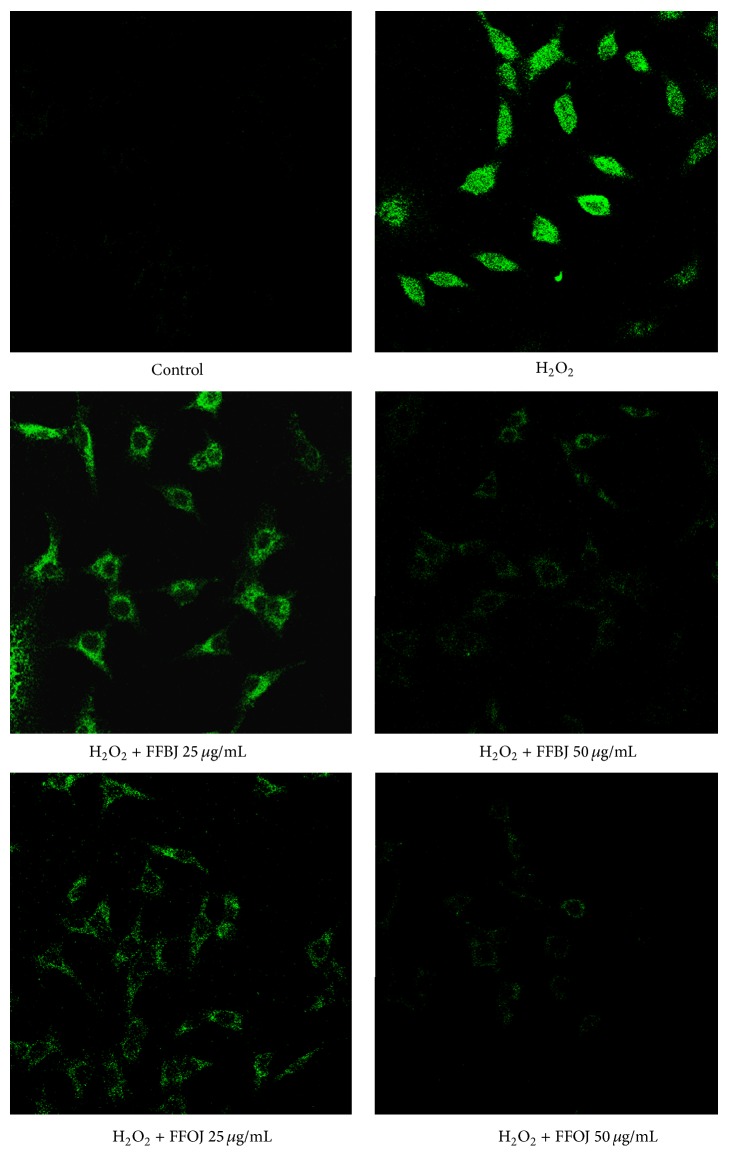
Confocal laser scanning microscope images of DCF-DA-stained cells. A549 cells grown on cell slides were preincubated with FFBJ or FFOJ and after 18 h exposed to H_2_O_2_ 200 *μ*M. Green fluorescence represented the amounts of ROS. Images shown are representative of three independent experiments. 400x magnification.

The data from FACS analyses were confirmed by CLSM observations.  Figure 4 shows that, in comparison to oxidatively stressed A549 cells, the presence of* Citrus *extracts dampened the fluorescence emission proportionally to the concentration employed.

**Figure 5:**
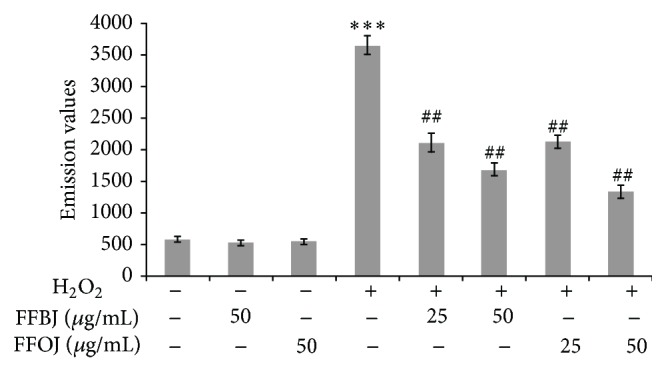
Cytofluorimetric evaluation of lipid hydroperoxides. The A549 cells incubated for 18 h with FFBJ or FFOJ were oxidatively stressed with H_2_O_2_ 200 *μ*M for 2 h and then loaded by DPPP probe. The graph reports the mean of fluorescence expressed in arbitrary fluorescence units (AFU) of three independent experiments. Data represent the mean ± SEM of at least three separate experiments. ^*∗∗∗*^ *P* < 0.001* versus* control cells and ^##^ *P* < 0.01* versus* H_2_O_2_-treated cells.

The high lipid content of cell membranes makes them particularly susceptible to oxidative damage. We therefore measured the lipid hydroperoxides to investigate the consequences of the oxidative damage caused by H_2_O_2_ on the membrane lipids and also to examine the effect of our extracts. The level of lipid peroxidation in A549 cells increased up to 6-fold after 2 h incubation with 200 *μ*M of H_2_O_2_, an effect counteracted by preincubation with either extract (Figure 5). Interestingly, the DPPP emission value detected in H_2_O_2_-stressed cells were lowered by up to 40 and 55% by pretreatment with FFBJ at concentrations of 25 and 50 *μ*g/mL, respectively (*P* < 0.01). Similar results were obtained using FFOJ (45 and 60% emission DPPP reduction by 25 and 50 *μ*g/mL concentrations, resp.; *P* < 0.01).

**Figure 6:**
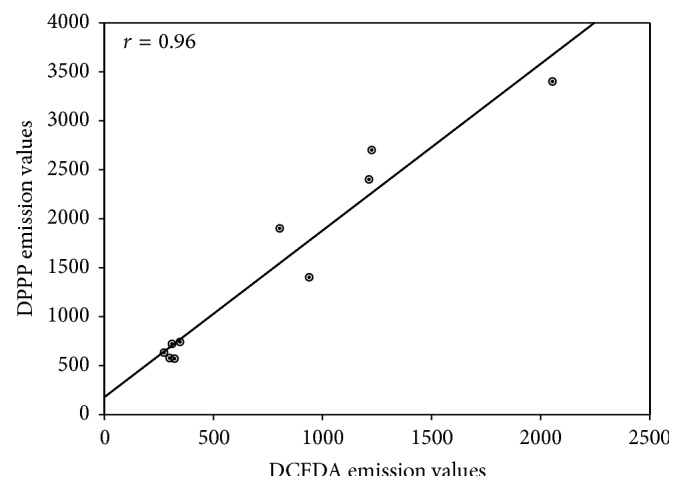
Pearson's correlation. Plot of DCF-DA* versus* DPPP emission values in A549 cells pretreated or not with FFBJ or FFOJ for 18 h and then incubated with H_2_O_2_ 200 *μ*M for additional 2 h. *r* = 0.96; *P* < 0.001.

The strong correlation between redox imbalance and lipid hydroperoxides in H_2_O_2_-stressed cells (*r* = 0.96; *P* < 0.001) strengthens the evidence supporting the potential of both extracts to counteract lipid peroxidation (Figure 6).

**Figure 7:**
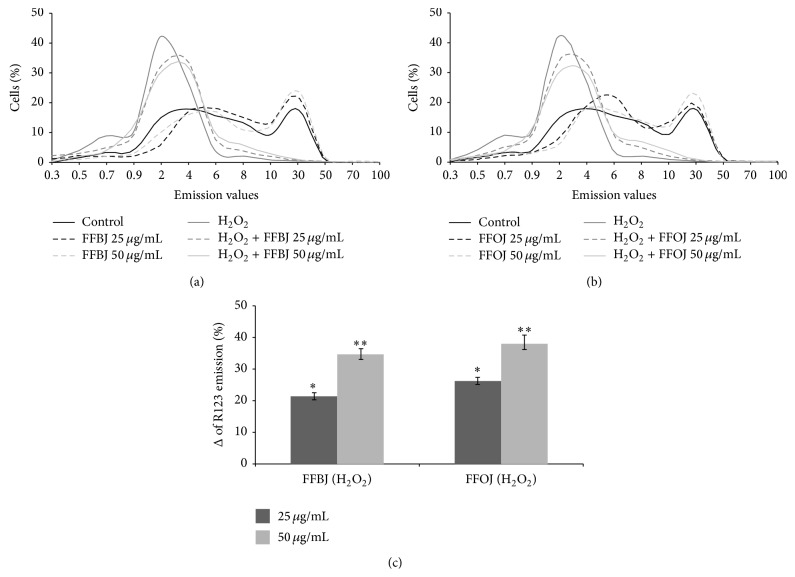
Effect of FFBJ or FFOJ on mitochondrial membrane potential in H_2_O_2_-treated cells. The cells treated with FFBJ or FFOJ and then exposed to 200 *μ*M H_2_O_2_ for 2 h were loaded with R123 probe. Fluorescence was followed by flow cytometry. The shift of the curves to the left of the graph indicates the reduction of Δ*ψ*_m_. Results from FFBJ (a) or FFOJ (b) exposure. In (c) are reported data from (a) and (b) expressed as percentage of increase (%Δ) of R123 emission values in FFBJ- or FFOJ-pretreated cells subsequently exposed to H_2_O_2 _compared to H_2_O_2_-stressed cells. ^*∗*^ *P* < 0.05 and ^*∗∗*^ *P* < 0.01* versus* H_2_O_2_-treated cells.

Since mitochondrial membrane phospholipids are key targets for lipid peroxidation involving highly polyunsaturated side chains, we further evaluated the ability of both extracts to restrain mitochondrial impairment.  Figure 7 shows that incubation of A549 cells with H_2_O_2_ caused a drop in Δ*ψ*_m_, as indicated by the peak of the R123 probe fluorescence emission on the left of the graph (Figures 7(a) and 7(b)). The presence of FFBJ or FFOJ lessened the ROS-induced mitochondrial impairment observed in stressed cells, reducing the number of cells with lower R123 fluorescence emission values. This is clearly shown in Figure 7(c) which presents the increases in weighted-averages fluorescence found in cultures preincubated with the* Citrus* extracts prior to H_2_O_2_ incubation, in comparison to the non-pretreated oxidatively stressed cells, set at 0 (*P* < 0.05 and *P* < 0.01 for 25 and 50 *μ*g/mL of both extracts, resp.).

**Figure 8:**
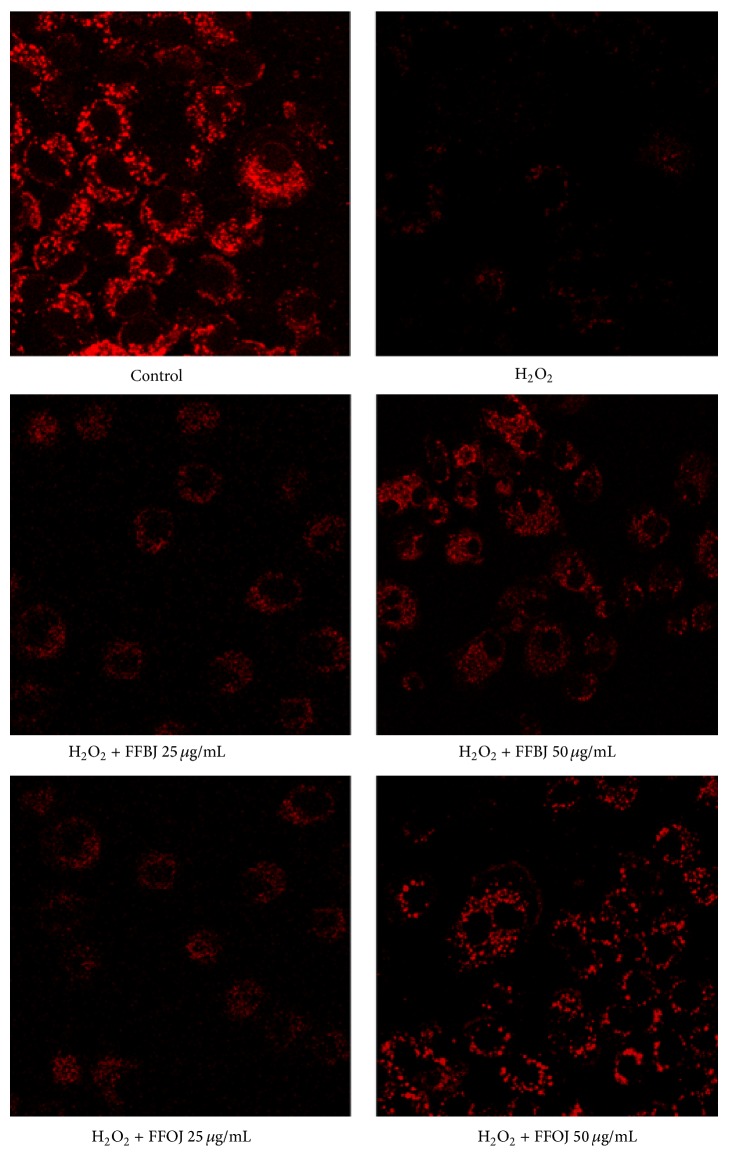
CLSM analysis of mitochondrial membrane potential. A549 cells were grown on cell slides and after treatment with FFBJ or FFOJ were incubated with H_2_O_2_. Mitochondrial membrane potential was detected by R123 staining. Red fluorescence indicates functional mitochondria. Images captured at 400x magnification are shown as representative from three independent experiments.

The CLSM observations confirmed the results from FACS analyses (Figure 8), showing that the presence of FFBJ or FFOJ reduced the fall in Δ*ψ*_m_, as revealed by the increased emitted fluorescence.

**Figure 9:**
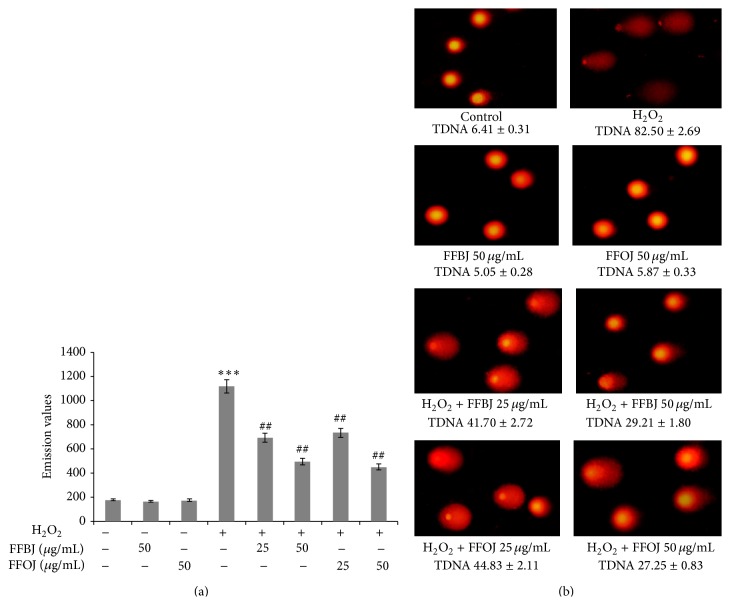
Protective effects of FFBJ and FFOJ on DNA damage induced by H_2_O_2_. (a) Levels of 8-oxo-dG are measured as emission signals of fluorochrome FITC-labeled avidin collected in the FL-1 channel. The graph reports the mean of fluorescence expressed in arbitrary fluorescence units (AFU) of three independent experiments. ^*∗∗∗*^ *P* < 0.001* versus* control cultures and ^##^ *P* < 0.01* versus* H_2_O_2_-treated cells. (b) Images of comet assay captured by fluorescence microscopy at a magnification of 400x. A representative experiment that was replicated three times with similar results is shown. Percentages of tail DNA (TDNA) are indicated.

Levels of 8-oxo-dG and DNA strand breaks were measured to study the effectiveness of the* Citrus* extracts to restrain DNA-oxidative damage. The results of cytofluorimetric analysis using a FITC-labelled avidin probe are reported in Figure 9. This shows that 18 h of treatment with FFBJ or FFOJ did not induce DNA oxidation since the emission values roughly overlapped those recorded in control cells. Despite the massive DNA damage induced by 2 h of H_2_O_2_ 200 *μ*M incubation, the pretreatment with FFBJ or FFOJ significantly decreased oxidative DNA damage by about 1.5 fold (*P* < 0.01; Figure 9(a)). Similar results were obtained using the comet assay (Figure 9(b)).

## 4. Discussion

The pathogenic role of oxidative damage in chronic-degenerative diseases has escalated scientific interest in the potential use of different natural drugs for prevention and/or as adjuvant therapy. In this study we investigated the effects of flavonoids extracted from orange and bergamot juices on H_2_O_2_-induced oxidative stress in human alveolar type II epithelial A549 cells, which resemble the pathophysiological lung conditions of respiratory epithelium [39]. A549 cells have been extensively used to gain insight into the cellular and molecular mechanisms of pulmonary diseases because they retain the characteristics of alveolar cells [40]. Alveolar cells have the capacity to modify the inflammatory reaction within the alveolar space. In particular, type II alveolar epithelial cells control the volume and composition of the epithelial lining fluid, and, in the event of injury, they differentiate into type I alveolar epithelial cells to maintain the integrity of the alveolar wall.

Hydrogen peroxide, widely used in* in vitro* models of oxidative stress, is a physiological constituent of living cells and is continuously produced* via* diverse cellular pathways. Its intracellular steady-state concentration is controlled by various enzymatic and nonenzymatic antioxidant systems and is assumed to vary between 1 and 700 nM [41]. Although H_2_O_2_ at physiological concentrations acts as a signaling molecule by modulating the expression of defense genes, levels above 1 *μ*M cause redox imbalance, inducing growth arrest and cell death [42]. Moreover, in Fenton-like reactions, H_2_O_2_ contributes to the formation of the hydroxyl radical (^∙^OH) in the presence of free redox-active transition metals, amplifying cellular damage. Due to its high reactivity and very short half life (in aqueous solution less than 1 ns), ^∙^OH is able to attack DNA, lipids, and proteins, more efficiently than other ROS.

The results of the present study suggest that, despite their qualitative and quantitative differences in the phenolic compounds content of FFBJ and FFOJ, both exert a significant and similar antioxidant effect on H_2_O_2_ cell injury. Indeed, although both bergamot and orange belong to the* Citrus* family, the characteristic flavonoid profile of their juices can influence their antioxidant properties. Significant differences were found between FFBJ and FFOJ, with higher activity observed for FFBJ extract than for FFOJ in a number of abiotic* in vitro *tests, including the ORAC, the Folin-Ciocalteu, and the Reducing Power assays. The latter two tests were carried out in alkaline and acid conditions, respectively. We used both tests to evaluate the antioxidant potential of polyphenolic compounds because an acidic pH may reduce* in vitro* phenolic antioxidant activity as a result of protonation, whereas an alkaline pH could enhance this activity [43]. Overall, with the exception of the DPPH test, all the cell-free assays provide evidence of a higher antioxidant activity of FFBJ compared to FFOJ.

Biological experiments were performed on A549 cells stressed by H_2_O_2_ injury. In this* in vitro* model, FFBJ behaved similarly to FFOJ, demonstrating an analogous antioxidant efficacy of both extracts. This observation, however, is quite in contrast to the results of tests performed in the cell-free models. We demonstrated that FFBJ and FFOJ were not only able to reduce ROS production, lipid hydroperoxides, and DNA damage induced by H_2_O_2_ in A549 cells, but also able to increase Δ*ψ*_m_.

Several studies have examined the antioxidant activity of flavonoids by cell-free tests alone, further supporting the structure-functional relationship [44–46]. Very recently, Lago et al. reviewed the effects of different flavonoids in certain lung disease and the structure-activity relationships, linking the biological potential and the chemical profile of these compounds [47]. Moreover, there are a range of studies that have assayed the antioxidant properties of natural drugs or single molecules in abiotic,* in vitro*, or* in vivo* models [48, 49]. However, few studies have been done which concomitantly analyse the antioxidant ability of two or more natural drugs both in cell-free and in cell-based assays [50–52]. The slight discrepancy between the results from chemical and biological assays found in this study could be due merely to the different experimental models employed. Indeed, while the chemical-based tests were performed under tightly controlled and, often, nonphysiological conditions, the biological ones were carried out in a more complex system (the cellular environment) and this may have influenced the experimental results. Moreover, antioxidant action is not limited to scavenging free radicals but includes the modulation of redox cell signaling and gene expression that greatly contribute to cellular antioxidant capacity. In addition, metabolic modifications occurring in cultured cells may substantially influence the antioxidant activity of flavonoids. In this regard, it should be stressed that A549 cells possess phases I and II enzymes capable of metabolizing xenobiotics [39, 53], which may help to explain the overlap in the antioxidant activity of FFBJ and FFOJ found in the cell-based assays in contrast to the more active performance of FFBJ than of FFOJ found in the cell-free models. It is therefore not surprising that differences emerged between the results from the chemical and biological tests, reported herein. Therefore, extreme caution is advised in extrapolating the performance of antioxidants from a cell-free assay to biological situations, and it is prudent to use more than a single method to evaluate the antioxidant properties of natural products. Our observations are consistent with the more recent literature, which supports the better predictive capacity of biological assays compared to chemical ones in order to evaluate the potential antioxidant activity of a compound or extract [52]. Chemical-based methods are useful for screening, because they are quick, easy to do, and cheap and have high-throughput and generally yield an index value that allows different products to be readily compared and ranked, while the cell-based assay are more appropriate to study the behaviour of an antioxidant under physiological conditions [50]. However, studies on animal models and human are necessary to assess the effectiveness of natural compounds. Indeed, it has been reported that flavonoids are extensively metabolised* in vivo* (especially when given orally) resulting in a significant alteration in their redox potentials.

Oxidative stress is the main pathological mechanism in lung disease and is directly linked to oxidation of proteins, DNA and lipids, mitochondrial impairment, and compromised lung defense mechanisms. H_2_O_2_ is an important marker of oxidative stress that can be generated by the xanthine/xanthine oxidase reaction, with higher amounts found in cell-free bronchoalveolar lavage fluid and plasma taken from both asthma and COPD patients, suggesting a central role in their pathogenesis [2]. This observation is strengthened by the evidence that patients with lung diseases are characterized by high concentrations of exhaled hydrogen peroxide, which further increases as the disease exacerbates [2]. Moreover, lipid peroxidation byproducts, including 8-isoprostane and hydrocarbons, (e.g., ethane and pentane) are also detectable in the air exhaled by these patients [54, 55], suggesting that membrane lipid peroxidation represents a further key event implicated in lung disorders. The results of our study demonstrated that both FFBJ and FFOJ counteract ROS generation induced by H_2_O_2_ in A549 cells and prevent lipidic hydroperoxides generation, which are precursors of malondialdehyde (MDA) and 4-hydroxynonenal (4-HNE), these being end-products of lipid peroxidation capable of inactivating antioxidant enzymes and causing redox imbalance. In addition, MDA may form adducts with DNA bases, while 4-HNE produces exocyclic etheno-DNA-base adducts [56].

The main effect of lipid peroxidation is the dysfunction of mitochondrial membranes determining energy failure and a number of intracellular events leading to cell death. In a recent study we demonstrated that exposure of A549 cells to airborne metals causes mitochondrial impairment due to lipid peroxidation of polyunsaturated fatty acids in membrane phospholipids amplifying oxidative stress [35]. It is known that the phospholipids of the inner mitochondrial membrane play a key role in optimizing the activity of mitochondrial proteins including several anion carriers, ADP/ATP translocators and some electron transport complexes. Due to the double bonds of their fatty acid constituents, mitochondrial phospholipids are particularly susceptible to peroxidative attack producing organelle impairment. Moreover, given that modified electron transport complexes cause a further increase of redox imbalance by endogenous ROS overproduction, a vicious circle is triggered, amplifying the oxidative cellular damage [57, 58]. Interestingly, the* Citrus* extracts used in this study reduced the drop in Δ*ψ*_m_ induced by H_2_O_2_ in A549 cells, thus restoring mitochondrial functions.

## 5. Conclusions

Our study demonstrates that the antioxidant properties of the flavonoid mixtures present in both FFBJ and FFOJ are able to inhibit the prooxidant effects of H_2_O_2_ on lung epithelial cells. These findings support the role of bergamot and orange flavonoids in the treatment of oxidative stress-related disorders and pave the way toward novel therapeutic approaches to protect against oxidative injury in lung diseases, a goal potentially achievable after evaluating the effectiveness of these extracts* in vivo.*

## Supplementary Material

Schematic illustration of the protection mechanism provided by FFBJ and FFOJ against H_2_O_2_-induced oxidative stress in human lung epithelial A549 cells.

## Figures and Tables

**Table 1:** Concentration of flavonoids identified in both FFBJ and FFOJ, expressed in mg/g of dried extract.

FFBJ		FFOJ	
Compound	mg/g	Compound	mg/g
Vicenin-2	11.61 ± 0.38	Vicenin-2	43.34 ± 0.50
Lucenin-2 4′-methyl ether	10.29 ± 1.24	Lucenin-2 4′-methyl ether	22.20 ± 1.10
Eriocitrin	8.89 ± 0.49	Neohesperidin	9.95 ± 0.98
Neoeriocitrin	51.73 ± 2.33	Eriocitrin	2.20 ± 0.70
Poncirin	18.41 ± 0.20	Narirutin	89.55 ± 4.11
Orientin 4′-methylether	14.85 ± 2.10	Neodiosmin	1.18 ± 2.14
Naringin	91.90 ± 1.82	Hesperidin	231.98 ± 6.65
Rhoifolin	19.96 ± 0.74	Didymin	15.97 ± 3.14
Hesperidin	7.49 ± 0.31	Sinensetin	3.28 ± 0.49
Isoquercitrin	2.55 ± 0.34	Tangeretin	7.03 ± 0.37
Neohesperidin	95.33 ± 2.00	Nobiletin	15.09 ± 1.31
Neodiosmin	12.39 ± 2.67		
Narirutin	4.97 ± 0.89		
Melitidin	79.47 ± 1.15		
Brutieridin	14.73 ± 1.38		
Naringenin	41.48 ± 0.20		
Hesperetin	53.84 ± 0.27		
Diosmetin	12.36 ± 2.09		

**Table 2:** Antioxidant activity of FFBJ and FFOJ evaluated by chemical tests. Results are reported as mean ± SEM of three experiments performed in triplicate and expressed in standard equivalent/g of dried extract. Data were analyzed by Student's *t*-test for unpaired data. ^*∗*^ *P* < 0.05, ^*∗∗*^ *P* < 0.01, and ^*∗∗∗*^ *P* < 0.001.

	FFBJ	FFOJ
ORAC *μ*mol TE/g	8054.17 ± 606.11	5166.05 ± 205.68^**∗∗**^
DPPH mg TE/g	74.5 ± 10.8	68.3 ± 4.05
Folin-Ciocalteu mg GAE/g	129.17 ± 2	122.8 ± 3.3^**∗**^
Reducing power mg AAE/g	99.05 ± 2	62.6 ± 3.1^**∗∗∗**^

## Abbreviations

AAE: Ascorbic acid equivalent
AFU: Arbitrary fluorescence units
AAPH: 2,2′-Azobis(2-amidinopropane) dihydrochloride
ARDS: Adult respiratory distress syndrome
BJe: Bergamot juice extract
CLSM: Confocal laser scanning microscopy
COPD: Chronic obstructive pulmonary disease
DCF-DA: 2-7-Dichlorofluorescein diacetate
DPPH: 2,2-Diphenylpicrylhydrazyl
DPPP: Diphenyl-1-pyrenylphosphine
Δ*ψ*_m_: Transmembrane mitochondrial potential
FACS: Fluorescence-activated cell sorting
FBS: Foetal bovine serum
FFBJ: Flavonoid fraction of bergamot juice
FFOJ: Flavonoid fraction of orange juice
GAE: Gallic acid equivalents
8-OH-dG: 8-Hydroxy-2′-deoxyguanosine
ORAC: Oxygen radical scavenging capacity
ROS: Reactive oxygen species
TE: Trolox equivalents.
