# Supplementary figures and images for: Flavonoid Fraction of Orange and Bergamot Juices Protect Human Lung Epithelial Cells from Hydrogen Peroxide-Induced Oxidative Stress
Source: Evid Based Complement Alternat Med. 2015 Jun 21;2015:957031. doi: 10.1155/2015/957031 (PMC4499611; doi:10.1155/2015/957031)

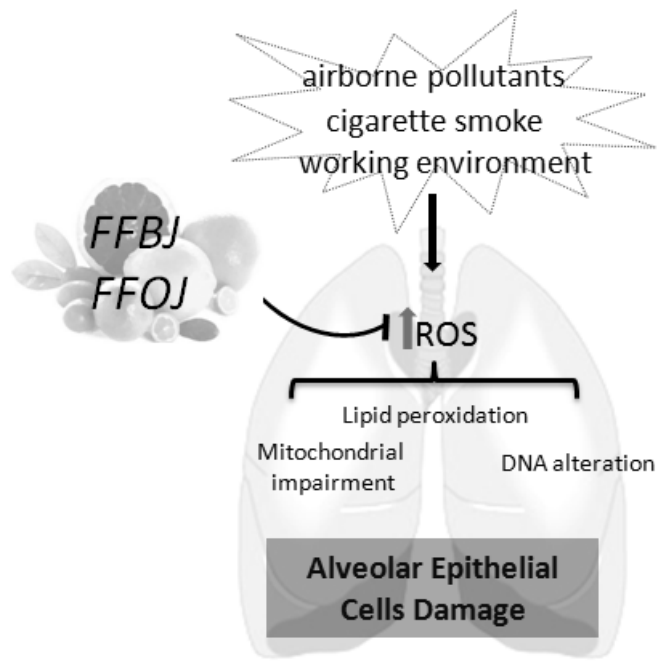

Supplement: Supplementary file 1 — Schematic illustration of the protection mechanism provided by FFBJ and FFOJ against H2O2-induced oxidative stress in human lung epithelial A549 cells. [file 957031.f1.pdf]
